# Supplementary material for: Factors influencing the effectiveness of artificial intelligence-assisted decision-making in medicine: a scoping review
Source: J Am Med Inform Assoc. 2026 Feb 20;33(5):1054–64. doi: 10.1093/jamia/ocag002 (PMC13127661; doi:10.1093/jamia/ocag002)
Supplement: ocag002_Supplementary_Data [file ocag002_supplementary_data.docx]

**Supplementary Materials**

**Factors Influencing the Effectiveness of AI-Assisted Decision-Making in Medicine: A Scoping Review**

**Table of Contents**

[**Supplementary Table 1. PRISCMA-ScR Checklist** 1](#_Toc213406906)

[**Supplementary Table 2. List of Included Studies** 4](#_Toc213406907)

[**Citations for Included Studies** 7](#_Toc213406908)

[**Queries used for study identification** 11](#_Toc213406909)

[**Extracted Data Fields** 14](#_Toc213406910)

# **Supplementary Table 1. PRISCMA-ScR Checklist**

**Preferred Reporting Items for Systematic reviews and Meta-Analyses extension for Scoping Reviews (PRISMA-ScR) Checklist**

| **SECTION** | **ITEM** | **PRISMA-ScR CHECKLIST ITEM** | **REPORTED ON PAGE #** |
| --- | --- | --- | --- |
| **TITLE** | | | |
| Title | 1 | Identify the report as a scoping review. | 1 |
| **ABSTRACT** | | | |
| Structured summary | 2 | Provide a structured summary that includes (as applicable): background, objectives, eligibility criteria, sources of evidence, charting methods, results, and conclusions that relate to the review questions and objectives. | 1 |
| **INTRODUCTION** | | | |
| Rationale | 3 | Describe the rationale for the review in the context of what is already known. Explain why the review questions/objectives lend themselves to a scoping review approach. | 2 |
| Objectives | 4 | Provide an explicit statement of the questions and objectives being addressed with reference to their key elements (e.g., population or participants, concepts, and context) or other relevant key elements used to conceptualize the review questions and/or objectives. | 2 |
| **METHODS** | | | |
| Protocol and registration | 5 | Indicate whether a review protocol exists; state if and where it can be accessed (e.g., a Web address); and if available, provide registration information, including the registration number. | 2 |
| Eligibility criteria | 6 | Specify characteristics of the sources of evidence used as eligibility criteria (e.g., years considered, language, and publication status), and provide a rationale. | 2 |
| Information sources* | 7 | Describe all information sources in the search (e.g., databases with dates of coverage and contact with authors to identify additional sources), as well as the date the most recent search was executed. | 2 |
| Search | 8 | Present the full electronic search strategy for at least 1 database, including any limits used, such that it could be repeated. | 2, Supplementary Materials p8 |
| Selection of sources of evidence† | 9 | State the process for selecting sources of evidence (i.e., screening and eligibility) included in the scoping review. | 2 |
| Data charting process‡ | 10 | Describe the methods of charting data from the included sources of evidence (e.g., calibrated forms or forms that have been tested by the team before their use, and whether data charting was done independently or in duplicate) and any processes for obtaining and confirming data from investigators. | 2 |
| Data items | 11 | List and define all variables for which data were sought and any assumptions and simplifications made. | 2 |
| Critical appraisal of individual sources of evidence§ | 12 | If done, provide a rationale for conducting a critical appraisal of included sources of evidence; describe the methods used and how this information was used in any data synthesis (if appropriate). | N/A |
| Synthesis of results | 13 | Describe the methods of handling and summarizing the data that were charted. | 2, OSF Repository (see README.txt) |
| **RESULTS** | | | |
| Selection of sources of evidence | 14 | Give numbers of sources of evidence screened, assessed for eligibility, and included in the review, with reasons for exclusions at each stage, ideally using a flow diagram. | 2-3 |
| Characteristics of sources of evidence | 15 | For each source of evidence, present characteristics for which data were charted and provide the citations. | Supplementary Materials p4. OSF Repository (Extracted_Processed.csv, Extracted_Factors_Results.csv) |
| Critical appraisal within sources of evidence | 16 | If done, present data on critical appraisal of included sources of evidence (see item 12). | N/A |
| Results of individual sources of evidence | 17 | For each included source of evidence, present the relevant data that were charted that relate to the review questions and objectives. | OSF Repository (Extracted_Processed.csv, Extracted_Factors_Results.csv) |
| Synthesis of results | 18 | Summarize and/or present the charting results as they relate to the review questions and objectives. | 3-6 |
| **DISCUSSION** | | | |
| Summary of evidence | 19 | Summarize the main results (including an overview of concepts, themes, and types of evidence available), link to the review questions and objectives, and consider the relevance to key groups. | 6-7 |
| Limitations | 20 | Discuss the limitations of the scoping review process. | 7 |
| Conclusions | 21 | Provide a general interpretation of the results with respect to the review questions and objectives, as well as potential implications and/or next steps. | 7-8 |
| **FUNDING** | | | |
| Funding | 22 | Describe sources of funding for the included sources of evidence, as well as sources of funding for the scoping review. Describe the role of the funders of the scoping review. | 8 |

JBI = Joanna Briggs Institute; PRISMA-ScR = Preferred Reporting Items for Systematic reviews and Meta-Analyses extension for Scoping Reviews.

* Where *sources of evidence* (see second footnote) are compiled from, such as bibliographic databases, social media platforms, and Web sites.

† A more inclusive/heterogeneous term used to account for the different types of evidence or data sources (e.g., quantitative and/or qualitative research, expert opinion, and policy documents) that may be eligible in a scoping review as opposed to only studies. This is not to be confused with *information sources* (see first footnote).

‡ The frameworks by Arksey and O’Malley (6) and Levac and colleagues (7) and the JBI guidance (4, 5) refer to the process of data extraction in a scoping review as data charting*.*

§ The process of systematically examining research evidence to assess its validity, results, and relevance before using it to inform a decision. This term is used for items 12 and 19 instead of "risk of bias" (which is more applicable to systematic reviews of interventions) to include and acknowledge the various sources of evidence that may be used in a scoping review (e.g., quantitative and/or qualitative research, expert opinion, and policy document).
*From:* Tricco AC, Lillie E, Zarin W, O'Brien KK, Colquhoun H, Levac D, et al. PRISMA Extension for Scoping Reviews (PRISMAScR): Checklist and Explanation. Ann Intern Med. 2018;169:467–473. [doi: 10.7326/M18-0850](http://annals.org/aim/fullarticle/2700389/prisma-extension-scoping-reviews-prisma-scr-checklist-explanation).

# **Supplementary Table 2. List of Included Studies**

| **Authors and Year** | **Title** | **Sample Size** | **Factors Studied** | **Outcomes Measured** |
| --- | --- | --- | --- | --- |
| Adam et al. 2022 | Mitigating the impact of biased artificial intelligence in emergency decision-making. | 954 | communication style | actions |
| Bond et al. 2018 | Automation bias in medicine: The influence of automated diagnoses on interpreter accuracy and uncertainty when reading electrocardiograms. | 30 | correctness of AI and expertise | attitudes |
| Cabitza et al. 2019 | Biases Affecting Human Decision Making in AI-Supported Second Opinion Settings | 75 | baseline attitudes and expertise | attitudes |
| Cabitza et al. 2023 | Rams, hounds and white boxes: Investigating human-AI collaboration protocols in medical diagnosis. | 56 | expertise, explainable AI, and order of information | performance |
| Calisto et al. 2022 | BreastScreening-AI: Evaluating medical intelligent agents for human-AI interactions. | 45 | expertise and explainable AI | attitudes and actions |
| Calisto et al. 2025 | Personalized explanations for clinician-AI interaction in breast imaging diagnosis by adapting communication to expertise levels | 52 | communication style | attitudes and performance |
| Carmichael et al. 2024 | Diagnostic decisions of specialist optometrists exposed to ambiguous deep-learning outputs. | 30 | expertise and explainable AI | attitudes, actions, and performance |
| Chen et al. 2024 | Trust in Machine Learning Driven Clinical Decision Support Tools Among Otolaryngologists. | 45 | baseline attitudes, expertise, and explainable AI | actions |
| deOliveira et al. 2025 | Effect of Explainable Artificial Intelligence on Trust of Mental Health Professionals in an AI-Based System for Suicide Prevention | 78 | AI knowledge, correctness of AI, expertise, and explainable AI | attitudes |
| Dorr et al. 2020 | COVID-19 pneumonia accurately detected on chest radiographs with artificial intelligence. | 54 | expertise | performance |
| Festor et al. 2025 | Safety of human-AI cooperative decision-making within intensive care: A physical simulation study | 38 | correctness of AI | actions |
| Fritz et al. 2024 | Effect of machine learning models on clinician prediction of postoperative complications: the Perioperative ORACLE randomised clinical trial | 89 | correctness of AI and expertise | performance |
| Gaube et al. 2021 | Do as AI say: susceptibility in deployment of clinical decision-aids. | 265 | baseline attitudes, correctness of AI, and expertise | attitudes and performance |
| Gaube et al. 2023 | Non-task expert physicians benefit from correct explainable AI advice when reviewing X-rays. | 223 | baseline attitudes, expertise, and explainable AI | attitudes, actions, and performance |
| Goel et al. 2022 | The effect of machine learning explanations on user trust for automated diagnosis of COVID-19. | 30 | explainable AI | attitudes |
| Goh et al. 2024 | Large Language Model Influence on Diagnostic Reasoning: A Randomized Clinical Trial | 50 | AI knowledge and expertise | performance |
| Gombolay et al. 2024 | Effects of explainable artificial intelligence in neurology decision support. | 365 | expertise and explainable AI | attitudes and performance |
| Gomez et al. 2024 | Explainable AI decision support improves accuracy during telehealth strep throat screening | 121 | expertise and explainable AI | attitudes, actions, and performance |
| Groh et al. 2024 | Deep learning-aided decision support for diagnosis of skin disease across skin tones. | 1118 | correctness of AI and expertise | performance |
| Guo et al. 2024 | Deep Learning for Chest X-ray Diagnosis: Competition Between Radiologists with or Without Artificial Intelligence Assistance. | 111 | expertise | performance |
| Jabbour et al. 2023 | Measuring the Impact of AI in the Diagnosis of Hospitalized Patients: A Randomized Clinical Vignette Survey Study. | 418 | correctness of AI, expertise, and explainable AI | performance |
| Jacobs et al. 2021 | How machine-learning recommendations influence clinician treatment selections: the example of the antidepressant selection. | 220 | AI knowledge, correctness of AI, and explainable AI | attitudes and performance |
| Jain et al. 2021 | Development and Assessment of an Artificial Intelligence-Based Tool for Skin Condition Diagnosis by Primary Care Physicians and Nurse Practitioners in Teledermatology Practices. | 40 | expertise | attitudes and performance |
| Jin et al. 2024 | Evaluating the clinical utility of artificial intelligence assistance and its explanation on the glioma grading task. | 35 | AI knowledge and explainable AI | attitudes, actions, and performance |
| Küper et al. 2025 | Psychological Factors Influencing Appropriate Reliance on AI-enabled Clinical Decision Support Systems: Experimental Web-Based Study Among Dermatologists | 223 | baseline attitudes, correctness of AI, and expertise | attitudes, actions, and performance |
| Knoery et al. 2019 | SPICED-ACS: Study of the potential impact of a computer-generated ECG diagnostic algorithmic certainty index in STEMI diagnosis: Towards transparent AI | 91 | expertise and uncertainty quantification | attitudes and performance |
| LancasterFarrell et al. 2022 | Explainability does not improve biochemistry staff trust in artificial intelligence-based decision support. | 159 | explainable AI | actions and performance |
| Laxar et al. 2023 | The influence of explainable vs non-explainable clinical decision support systems on rapid triage decisions: a mixed methods study. | 32 | baseline attitudes, expertise, and explainable AI | attitudes and actions |
| Lee et al. 2023 | Effect of Human-AI Interaction on Detection of Malignant Lung Nodules on Chest Radiographs. | 30 | expertise | actions and performance |
| Li et al. 2022 | How does the artificial intelligence-based image-assisted technique help physicians in diagnosis of pulmonary adenocarcinoma? A randomized controlled experiment of multicenter physicians in China. | 104 | available time and baseline attitudes | performance |
| Li et al. 2025 | Understanding physicians' noncompliance use of AI-aided diagnosis-A mixed-methods approach | 160 | baseline attitudes | actions |
| Luan et al. 2025 | Machine Learning-Aided Diagnosis Enhances Human Detection of Perilunate Dislocations | 137 | expertise | performance |
| Maehara et al. 2025 | Artificial intelligence support improves diagnosis accuracy in anterior segment eye diseases | 40 | expertise | performance |
| Micocci et al. 2021 | Attitudes towards Trusting Artificial Intelligence Insights and Factors to Prevent the Passive Adherence of GPs: A Pilot Study. | 50 | baseline attitudes and correctness of AI | actions and performance |
| Nagendran et al. 2023 | Quantifying the impact of AI recommendations with explanations on prescription decision making. | 86 | baseline attitudes, expertise, and explainable AI | attitudes and actions |
| Prinster et al. 2024 | Care to Explain? AI Explanation Types Differentially Impact Chest Radiograph Diagnostic Performance and Physician Trust in AI | 220 | correctness of AI, expertise, explainable AI, and uncertainty quantification | attitudes and performance |
| Rainey et al. 2023 | An experimental machine learning study investigating the decision-making process of students and qualified radiographers when interpreting radiographic images. | 106 | expertise | attitudes |
| Senoner et al. 2024 | Explainable AI improves task performance in human-AI collaboration | 113 | explainable AI | actions and performance |
| Tschandl et al. 2020 | Human-computer collaboration for skin cancer recognition. | 456 | baseline attitudes, correctness of AI, expertise, and explainable AI | actions and performance |
| Wang et al. 2023 | Artificial intelligence suppression as a strategy to mitigate artificial intelligence automation bias. | 40 | expertise | actions and performance |
| Wang et al. 2024 | A Deep Learning Model Enhances Clinicians' Diagnostic Accuracy to More Than 96% for Anterior Cruciate Ligament Ruptures on Magnetic Resonance Imaging. | 38 | expertise and task difficulty | performance |
| Williams et al. 2024 | Artificial Intelligence Assisted Surgical Scene Recognition: A Comparative Study Amongst Healthcare Professionals | 348 | expertise | performance |
| Yoon et al. 2023 | Developing and Evaluating an AI-Based Computer-Aided Diagnosis System for Retinal Disease: Diagnostic Study for Central Serous Chorioretinopathy. | 66 | expertise and explainable AI | performance |
| Yu et al. 2024 | Heterogeneity and predictors of the effects of AI assistance on radiologists. | 140 | correctness of AI and expertise | performance |
| Zhang et al. 2025 | Evaluating the effectiveness of a clinical decision support system (AI-Antidelirium) to improve Nurses' adherence to delirium guidelines in the intensive care unit | 80 | expertise and longitudinality | performance |

# **Citations for Included Studies**

1. Adam H, Balagopalan A, Alsentzer E, Christia F, Ghassemi M. Mitigating the impact of biased artificial intelligence in emergency decision-making. Commun Med (Lond). 2022;2(1):149.

2. Bond RR, Novotny T, Andrsova I, Koc L, Sisakova M, Finlay D, et al. Automation bias in medicine: The influence of automated diagnoses on interpreter accuracy and uncertainty when reading electrocardiograms. J Electrocardiol. 2018;51(6S):S6–11.

3. Cabitza F. Biases Affecting Human Decision Making in AI-Supported Second Opinion Settings. In 2019. p. 283–94.

4. Cabitza F, Campagner A, Ronzio L, Cameli M, Mandoli GE, Pastore MC, et al. Rams, hounds and white boxes: Investigating human-AI collaboration protocols in medical diagnosis. Artif Intell Med. 2023;138:102506.

5. Calisto FM, Santiago C, Nunes N, Nascimento JC. BreastScreening-AI: Evaluating medical intelligent agents for human-AI interactions. Artif Intell Med. 2022;127:102285.

6. Calisto F, Abrantes J, Santiago C, Nunes N, Nascimento J. Personalized explanations for clinician-AI interaction in breast imaging diagnosis by adapting communication to expertise levels. INTERNATIONAL JOURNAL OF HUMAN-COMPUTER STUDIES. 2025;197.

7. Carmichael J, Costanza E, Blandford A, Struyven R, Keane PA, Balaskas K. Diagnostic decisions of specialist optometrists exposed to ambiguous deep-learning outputs. Sci Rep. 2024;14(1):6775.

8. Chen H, Ma X, Rives H, Serpedin A, Yao P, Rameau A. Trust in Machine Learning Driven Clinical Decision Support Tools Among Otolaryngologists. Laryngoscope. 2024;

9. de Oliveira A, Azevedo J, Ruback L, Moreira R, Teixeira S, Teles A. Effect of Explainable Artificial Intelligence on Trust of Mental Health Professionals in an AI-Based System for Suicide Prevention. IEEE ACCESS. 2025;13:60987–1005.

10. Dorr F, Chaves H, Serra MM, Ramirez A, Costa ME, Seia J, et al. COVID-19 pneumonia accurately detected on chest radiographs with artificial intelligence. Intell Based Med. 2020;3:100014.

11. Festor P, Nagendran M, Gordon A, Faisal A, Komorowski M. Safety of human-AI cooperative decision-making within intensive care: A physical simulation study. PLOS DIGITAL HEALTH. 2025;4(2).

12. Fritz BA, King CR, Abdelhack M, Chen Y, Kronzer A, Abraham J, et al. Effect of machine learning models on clinician prediction of postoperative complications: the Perioperative ORACLE randomised clinical trial. British Journal of Anaesthesia. 2024;133(5):1042–50.

13. Gaube S, Suresh H, Raue M, Merritt A, Berkowitz SJ, Lermer E, et al. Do as AI say: susceptibility in deployment of clinical decision-aids. NPJ Digit Med. 2021;4(1):31.

14. Gaube S, Suresh H, Raue M, Lermer E, Koch TK, Hudecek MFC, et al. Non-task expert physicians benefit from correct explainable AI advice when reviewing X-rays. Sci Rep. 2023;13(1):1383.

15. Goel K, Sindhgatta R, Kalra S, Goel R, Mutreja P. The effect of machine learning explanations on user trust for automated diagnosis of COVID-19. Comput Biol Med. 2022;146:105587.

16. Goh E, Gallo R, Hom J, Strong E, Weng Y, Kerman H, et al. Large Language Model Influence on Diagnostic Reasoning: A Randomized Clinical Trial. JAMA Network Open [Internet]. 2024;7(10). Available from: https://www.embase.com/search/results?subaction=viewrecord&id=L2038335996&from=export

17. Gombolay GY, Silva A, Schrum M, Gopalan N, Hallman-Cooper J, Dutt M, et al. Effects of explainable artificial intelligence in neurology decision support. Ann Clin Transl Neurol. 2024;

18. Gomez C, Smith B, Zayas A, Unberath M, Canares T. Explainable AI decision support improves accuracy during telehealth strep throat screening. COMMUNICATIONS MEDICINE. 2024;4(1).

19. Groh M, Badri O, Daneshjou R, Koochek A, Harris C, Soenksen LR, et al. Deep learning-aided decision support for diagnosis of skin disease across skin tones. Nat Med. 2024;30(2):573–83.

20. Guo L, Zhou C, Xu J, Huang C, Yu Y, Lu G. Deep Learning for Chest X-ray Diagnosis: Competition Between Radiologists with or Without Artificial Intelligence Assistance. J Imaging Inform Med. 2024;

21. Jabbour S, Fouhey D, Shepard S, Valley TS, Kazerooni EA, Banovic N, et al. Measuring the Impact of AI in the Diagnosis of Hospitalized Patients: A Randomized Clinical Vignette Survey Study. JAMA. 2023;330(23):2275–84.

22. Jacobs M, Pradier MF, McCoy TH Jr, Perlis RH, Doshi-Velez F, Gajos KZ. How machine-learning recommendations influence clinician treatment selections: the example of the antidepressant selection. Transl Psychiatry. 2021;11(1):108.

23. Jain A, Way D, Gupta V, Gao Y, de Oliveira Marinho G, Hartford J, et al. Development and Assessment of an Artificial Intelligence-Based Tool for Skin Condition Diagnosis by Primary Care Physicians and Nurse Practitioners in Teledermatology Practices. JAMA Netw Open. 2021;4(4):e217249.

24. Jin W, Fatehi M, Guo R, Hamarneh G. Evaluating the clinical utility of artificial intelligence assistance and its explanation on the glioma grading task. Artif Intell Med. 2024;148:102751.

25. Knoery CR, Bond R, Iftikhar A, Rjoob K, McGilligan V, Peace A, et al. SPICED-ACS: Study of the potential impact of a computer-generated ECG diagnostic algorithmic certainty index in STEMI diagnosis: Towards transparent AI. Journal of Electrocardiology. 2019;57((Knoery C.R., charles.knoery@uhi.ac.uk; Heaton J.; Leslie S.J.) Division of Rural Health and Wellbeing, University of Highlands and Islands, Inverness, United Kingdom(Bond R.; Iftikhar A.; Rjoob K.) Ulster University, Jordanstown Campus, Shore Rd, Newtown):S86–91.

26. Küper A, Lodde GC, Livingstone E, Schadendorf D, Krämer N. Psychological Factors Influencing Appropriate Reliance on AI-enabled Clinical Decision Support Systems: Experimental Web-Based Study Among Dermatologists. Journal of Medical Internet Research [Internet]. 2025;27((Küper A., alisa.kueper@uni-due.de; Krämer N.) Social Psychology: Media and Communication, University of Duisburg-Essen, Duisburg, Germany(Lodde G.C.; Livingstone E.; Schadendorf D.) Department of Dermatology, University Hospital Essen, Essen, Germany). Available from: https://www.embase.com/search/results?subaction=viewrecord&id=L2038219509&from=export

27. Lancaster Farrell CJ. Explainability does not improve biochemistry staff trust in artificial intelligence-based decision support. Ann Clin Biochem. 2022;59(6):447–9.

28. Laxar D, Eitenberger M, Maleczek M, Kaider A, Hammerle FP, Kimberger O. The influence of explainable vs non-explainable clinical decision support systems on rapid triage decisions: a mixed methods study. BMC Med. 2023;21(1):359.

29. Lee JH, Hong H, Nam G, Hwang EJ, Park CM. Effect of Human-AI Interaction on Detection of Malignant Lung Nodules on Chest Radiographs. Radiology. 2023;307(5):e222976.

30. Li J, Zhou L, Zhan Y, Xu H, Zhang C, Shan F, et al. How does the artificial intelligence-based image-assisted technique help physicians in diagnosis of pulmonary adenocarcinoma? A randomized controlled experiment of multicenter physicians in China. J Am Med Inform Assoc. 2022;29(12):2041–9.

31. Li J, Li X, Zhang C. Understanding physicians’ noncompliance use of AI-aided diagnosis-A mixed-methods approach. DECISION SUPPORT SYSTEMS. 2025;191.

32. Luan A, von Rabenau L, Serebrakian A, Crowe C, Do B, Eberlin K, et al. Machine Learning-Aided Diagnosis Enhances Human Detection of Perilunate Dislocations. HAND-AMERICAN ASSOCIATION FOR HAND SURGERY. 2025;

33. Maehara H, Ueno Y, Yamaguchi T, Kitaguchi Y, Miyazaki D, Nejima R, et al. Artificial intelligence support improves diagnosis accuracy in anterior segment eye diseases. SCIENTIFIC REPORTS. 2025;15(1).

34. Micocci M, Borsci S, Thakerar V, Walne S, Manshadi Y, Edridge F, et al. Attitudes towards Trusting Artificial Intelligence Insights and Factors to Prevent the Passive Adherence of GPs: A Pilot Study. J Clin Med. 2021;10(14).

35. Nagendran M, Festor P, Komorowski M, Gordon AC, Faisal AA. Quantifying the impact of AI recommendations with explanations on prescription decision making. NPJ Digit Med. 2023;6(1):206.

36. Prinster D, Mahmood A, Saria S, Jeudy J, Lin CT, Yi PH, et al. Care to Explain? AI Explanation Types Differentially Impact Chest Radiograph Diagnostic Performance and Physician Trust in AI. Radiology [Internet]. 2024;313(2). Available from: https://www.embase.com/search/results?subaction=viewrecord&id=L2036128647&from=export

37. Rainey C, Villikudathil AT, McConnell J, Hughes C, Bond R, McFadden S. An experimental machine learning study investigating the decision-making process of students and qualified radiographers when interpreting radiographic images. PLOS Digit Health. 2023;2(10):e0000229.

38. Senoner J, Schallmoser S, Kratzwald B, Feuerriegel S, Netland T. Explainable AI improves task performance in human-AI collaboration. SCIENTIFIC REPORTS. 2024;14(1).

39. Tschandl P, Rinner C, Apalla Z, Argenziano G, Codella N, Halpern A, et al. Human-computer collaboration for skin cancer recognition. Nat Med. 2020;26(8):1229–34.

40. Wang DY, Ding J, Sun AL, Liu SG, Jiang D, Li N, et al. Artificial intelligence suppression as a strategy to mitigate artificial intelligence automation bias. J Am Med Inform Assoc. 2023;30(10):1684–92.

41. Wang DY, Liu SG, Ding J, Sun AL, Jiang D, Jiang J, et al. A Deep Learning Model Enhances Clinicians’ Diagnostic Accuracy to More Than 96% for Anterior Cruciate Ligament Ruptures on Magnetic Resonance Imaging. Arthroscopy. 2024;40(4):1197–205.

42. Williams SC, Zhou J, Muirhead WR, Khan DZ, Koh CH, Ahmed R, et al. Artificial Intelligence Assisted Surgical Scene Recognition: A Comparative Study Amongst Healthcare Professionals. Annals of Surgery [Internet]. 2024;((Williams S.C., simon.williams32@nhs.net; Zhou J., zjf@umich.edu; Muirhead W.R., w.muirhead@ucl.ac.uk; Khan D.Z., d.khan@ucl.ac.uk; Koh C.H., austin.koh@ucl.ac.uk; Ahmed R., razna.ahmed.21@alumni.ucl.ac.uk; Funnell J.P., jonathan.funnell.13@ucl.ac.uk; Han). Available from: https://www.embase.com/search/results?subaction=viewrecord&id=L2035484291&from=export

43. Yoon J, Han J, Ko J, Choi S, Park JI, Hwang JS, et al. Developing and Evaluating an AI-Based Computer-Aided Diagnosis System for Retinal Disease: Diagnostic Study for Central Serous Chorioretinopathy. J Med Internet Res. 2023;25:e48142.

44. Yu F, Moehring A, Banerjee O, Salz T, Agarwal N, Rajpurkar P. Heterogeneity and predictors of the effects of AI assistance on radiologists. Nat Med. 2024;30(3):837–49.

45. Zhang S, Ding S, Cui W, Li X, Wei J, Wu Y. Evaluating the effectiveness of a clinical decision support system (AI-Antidelirium) to improve Nurses’ adherence to delirium guidelines in the intensive care unit. INTENSIVE AND CRITICAL CARE NURSING. 2025;87.

# **Queries used for study identification**

- This query was generated programmatically and used to search PubMed. The queries used to search Embase and WebOfScience are identical, but were changed to fit the syntax of their respective sites.
- For brevity, the queries used to search Embase and Web Of Science are located in the OSF Repository (<https://osf.io/un32b/?view_only=efbeaf42a9ef4aaab45a2cde69450265>) under “Lit_Review_Queries.txt”.

(("artificial intelligence"[tiab] OR "machine learning"[tiab] OR "deep learning"[tiab] OR "artificial-intelligence"[tiab] OR "machine-learning"[tiab] OR "deep-learning"[tiab] OR "AI"[tiab]) AND (("reader study"[tiab] OR "reader studies"[tiab] OR "vignette"[tiab] OR "vignettes"[tiab] OR "user study"[tiab] OR "usability study"[tiab]) OR ("human-AI"[tiab] OR "clinician-AI"[tiab] OR "physician-AI"[tiab] OR "physician-machine"[tiab] OR "AI based recommendation"[tiab] OR "AI based recommendations"[tiab] OR "AI based assistance"[tiab] OR "AI based support"[tiab] OR "AI based advice"[tiab] OR "AI based decision support"[tiab] OR "AI based diagnostic systems"[tiab] OR "AI based diagnosis"[tiab] OR "AI-based recommendation"[tiab] OR "AI-based recommendations"[tiab] OR "AI-based assistance"[tiab] OR "AI-based support"[tiab] OR "AI-based advice"[tiab] OR "AI-based decision support"[tiab] OR "AI-based diagnostic systems"[tiab] OR "AI-based diagnosis"[tiab] OR "AI recommendation"[tiab] OR "AI recommendations"[tiab] OR "AI assistance"[tiab] OR "AI support"[tiab] OR "AI advice"[tiab] OR "AI decision support"[tiab] OR "AI diagnostic systems"[tiab] OR "AI diagnosis"[tiab] OR "AI-recommendation"[tiab] OR "AI-recommendations"[tiab] OR "AI-assistance"[tiab] OR "AI-support"[tiab] OR "AI-advice"[tiab] OR "AI-decision support"[tiab] OR "AI-diagnostic systems"[tiab] OR "AI-diagnosis"[tiab] OR "AI-generated recommendation"[tiab] OR "AI-generated recommendations"[tiab] OR "AI-generated assistance"[tiab] OR "AI-generated support"[tiab] OR "AI-generated advice"[tiab] OR "AI-generated decision support"[tiab] OR "AI-generated diagnostic systems"[tiab] OR "AI-generated diagnosis"[tiab] OR "AI generated recommendation"[tiab] OR "AI generated recommendations"[tiab] OR "AI generated assistance"[tiab] OR "AI generated support"[tiab] OR "AI generated advice"[tiab] OR "AI generated decision support"[tiab] OR "AI generated diagnostic systems"[tiab] OR "AI generated diagnosis"[tiab] OR "AI-aided recommendation"[tiab] OR "AI-aided recommendations"[tiab] OR "AI-aided assistance"[tiab] OR "AI-aided support"[tiab] OR "AI-aided advice"[tiab] OR "AI-aided decision support"[tiab] OR "AI-aided diagnostic systems"[tiab] OR "AI-aided diagnosis"[tiab] OR "AI aided recommendation"[tiab] OR "AI aided recommendations"[tiab] OR "AI aided assistance"[tiab] OR "AI aided support"[tiab] OR "AI aided advice"[tiab] OR "AI aided decision support"[tiab] OR "AI aided diagnostic systems"[tiab] OR "AI aided diagnosis"[tiab] OR "machine learning based recommendation"[tiab] OR "machine learning based recommendations"[tiab] OR "machine learning based assistance"[tiab] OR "machine learning based support"[tiab] OR "machine learning based advice"[tiab] OR "machine learning based decision support"[tiab] OR "machine learning based diagnostic systems"[tiab] OR "machine learning based diagnosis"[tiab] OR "machine learning-based recommendation"[tiab] OR "machine learning-based recommendations"[tiab] OR "machine learning-based assistance"[tiab] OR "machine learning-based support"[tiab] OR "machine learning-based advice"[tiab] OR "machine learning-based decision support"[tiab] OR "machine learning-based diagnostic systems"[tiab] OR "machine learning-based diagnosis"[tiab] OR "machine learning recommendation"[tiab] OR "machine learning recommendations"[tiab] OR "machine learning assistance"[tiab] OR "machine learning support"[tiab] OR "machine learning advice"[tiab] OR "machine learning decision support"[tiab] OR "machine learning diagnostic systems"[tiab] OR "machine learning diagnosis"[tiab] OR "machine learning-recommendation"[tiab] OR "machine learning-recommendations"[tiab] OR "machine learning-assistance"[tiab] OR "machine learning-support"[tiab] OR "machine learning-advice"[tiab] OR "machine learning-decision support"[tiab] OR "machine learning-diagnostic systems"[tiab] OR "machine learning-diagnosis"[tiab] OR "machine learning-generated recommendation"[tiab] OR "machine learning-generated recommendations"[tiab] OR "machine learning-generated assistance"[tiab] OR "machine learning-generated support"[tiab] OR "machine learning-generated advice"[tiab] OR "machine learning-generated decision support"[tiab] OR "machine learning-generated diagnostic systems"[tiab] OR "machine learning-generated diagnosis"[tiab] OR "machine learning generated recommendation"[tiab] OR "machine learning generated recommendations"[tiab] OR "machine learning generated assistance"[tiab] OR "machine learning generated support"[tiab] OR "machine learning generated advice"[tiab] OR "machine learning generated decision support"[tiab] OR "machine learning generated diagnostic systems"[tiab] OR "machine learning generated diagnosis"[tiab] OR "machine learning-aided recommendation"[tiab] OR "machine learning-aided recommendations"[tiab] OR "machine learning-aided assistance"[tiab] OR "machine learning-aided support"[tiab] OR "machine learning-aided advice"[tiab] OR "machine learning-aided decision support"[tiab] OR "machine learning-aided diagnostic systems"[tiab] OR "machine learning-aided diagnosis"[tiab] OR "machine learning aided recommendation"[tiab] OR "machine learning aided recommendations"[tiab] OR "machine learning aided assistance"[tiab] OR "machine learning aided support"[tiab] OR "machine learning aided advice"[tiab] OR "machine learning aided decision support"[tiab] OR "machine learning aided diagnostic systems"[tiab] OR "machine learning aided diagnosis"[tiab] OR "deep learning based recommendation"[tiab] OR "deep learning based recommendations"[tiab] OR "deep learning based assistance"[tiab] OR "deep learning based support"[tiab] OR "deep learning based advice"[tiab] OR "deep learning based decision support"[tiab] OR "deep learning based diagnostic systems"[tiab] OR "deep learning based diagnosis"[tiab] OR "deep learning-based recommendation"[tiab] OR "deep learning-based recommendations"[tiab] OR "deep learning-based assistance"[tiab] OR "deep learning-based support"[tiab] OR "deep learning-based advice"[tiab] OR "deep learning-based decision support"[tiab] OR "deep learning-based diagnostic systems"[tiab] OR "deep learning-based diagnosis"[tiab] OR "deep learning recommendation"[tiab] OR "deep learning recommendations"[tiab] OR "deep learning assistance"[tiab] OR "deep learning support"[tiab] OR "deep learning advice"[tiab] OR "deep learning decision support"[tiab] OR "deep learning diagnostic systems"[tiab] OR "deep learning diagnosis"[tiab] OR "deep learning-recommendation"[tiab] OR "deep learning-recommendations"[tiab] OR "deep learning-assistance"[tiab] OR "deep learning-support"[tiab] OR "deep learning-advice"[tiab] OR "deep learning-decision support"[tiab] OR "deep learning-diagnostic systems"[tiab] OR "deep learning-diagnosis"[tiab] OR "deep learning-generated recommendation"[tiab] OR "deep learning-generated recommendations"[tiab] OR "deep learning-generated assistance"[tiab] OR "deep learning-generated support"[tiab] OR "deep learning-generated advice"[tiab] OR "deep learning-generated decision support"[tiab] OR "deep learning-generated diagnostic systems"[tiab] OR "deep learning-generated diagnosis"[tiab] OR "deep learning generated recommendation"[tiab] OR "deep learning generated recommendations"[tiab] OR "deep learning generated assistance"[tiab] OR "deep learning generated support"[tiab] OR "deep learning generated advice"[tiab] OR "deep learning generated decision support"[tiab] OR "deep learning generated diagnostic systems"[tiab] OR "deep learning generated diagnosis"[tiab] OR "deep learning-aided recommendation"[tiab] OR "deep learning-aided recommendations"[tiab] OR "deep learning-aided assistance"[tiab] OR "deep learning-aided support"[tiab] OR "deep learning-aided advice"[tiab] OR "deep learning-aided decision support"[tiab] OR "deep learning-aided diagnostic systems"[tiab] OR "deep learning-aided diagnosis"[tiab] OR "deep learning aided recommendation"[tiab] OR "deep learning aided recommendations"[tiab] OR "deep learning aided assistance"[tiab] OR "deep learning aided support"[tiab] OR "deep learning aided advice"[tiab] OR "deep learning aided decision support"[tiab] OR "deep learning aided diagnostic systems"[tiab] OR "deep learning aided diagnosis"[tiab] OR "artificial intelligence based recommendation"[tiab] OR "artificial intelligence based recommendations"[tiab] OR "artificial intelligence based assistance"[tiab] OR "artificial intelligence based support"[tiab] OR "artificial intelligence based advice"[tiab] OR "artificial intelligence based decision support"[tiab] OR "artificial intelligence based diagnostic systems"[tiab] OR "artificial intelligence based diagnosis"[tiab] OR "artificial intelligence-based recommendation"[tiab] OR "artificial intelligence-based recommendations"[tiab] OR "artificial intelligence-based assistance"[tiab] OR "artificial intelligence-based support"[tiab] OR "artificial intelligence-based advice"[tiab] OR "artificial intelligence-based decision support"[tiab] OR "artificial intelligence-based diagnostic systems"[tiab] OR "artificial intelligence-based diagnosis"[tiab] OR "artificial intelligence recommendation"[tiab] OR "artificial intelligence recommendations"[tiab] OR "artificial intelligence assistance"[tiab] OR "artificial intelligence support"[tiab] OR "artificial intelligence advice"[tiab] OR "artificial intelligence decision support"[tiab] OR "artificial intelligence diagnostic systems"[tiab] OR "artificial intelligence diagnosis"[tiab] OR "artificial intelligence-recommendation"[tiab] OR "artificial intelligence-recommendations"[tiab] OR "artificial intelligence-assistance"[tiab] OR "artificial intelligence-support"[tiab] OR "artificial intelligence-advice"[tiab] OR "artificial intelligence-decision support"[tiab] OR "artificial intelligence-diagnostic systems"[tiab] OR "artificial intelligence-diagnosis"[tiab] OR "artificial intelligence-generated recommendation"[tiab] OR "artificial intelligence-generated recommendations"[tiab] OR "artificial intelligence-generated assistance"[tiab] OR "artificial intelligence-generated support"[tiab] OR "artificial intelligence-generated advice"[tiab] OR "artificial intelligence-generated decision support"[tiab] OR "artificial intelligence-generated diagnostic systems"[tiab] OR "artificial intelligence-generated diagnosis"[tiab] OR "artificial intelligence generated recommendation"[tiab] OR "artificial intelligence generated recommendations"[tiab] OR "artificial intelligence generated assistance"[tiab] OR "artificial intelligence generated support"[tiab] OR "artificial intelligence generated advice"[tiab] OR "artificial intelligence generated decision support"[tiab] OR "artificial intelligence generated diagnostic systems"[tiab] OR "artificial intelligence generated diagnosis"[tiab] OR "artificial intelligence-aided recommendation"[tiab] OR "artificial intelligence-aided recommendations"[tiab] OR "artificial intelligence-aided assistance"[tiab] OR "artificial intelligence-aided support"[tiab] OR "artificial intelligence-aided advice"[tiab] OR "artificial intelligence-aided decision support"[tiab] OR "artificial intelligence-aided diagnostic systems"[tiab] OR "artificial intelligence-aided diagnosis"[tiab] OR "artificial intelligence aided recommendation"[tiab] OR "artificial intelligence aided recommendations"[tiab] OR "artificial intelligence aided assistance"[tiab] OR "artificial intelligence aided support"[tiab] OR "artificial intelligence aided advice"[tiab] OR "artificial intelligence aided decision support"[tiab] OR "artificial intelligence aided diagnostic systems"[tiab] OR "artificial intelligence aided diagnosis"[tiab] OR "ML based recommendation"[tiab] OR "ML based recommendations"[tiab] OR "ML based assistance"[tiab] OR "ML based support"[tiab] OR "ML based advice"[tiab] OR "ML based decision support"[tiab] OR "ML based diagnostic systems"[tiab] OR "ML based diagnosis"[tiab] OR "ML-based recommendation"[tiab] OR "ML-based recommendations"[tiab] OR "ML-based assistance"[tiab] OR "ML-based support"[tiab] OR "ML-based advice"[tiab] OR "ML-based decision support"[tiab] OR "ML-based diagnostic systems"[tiab] OR "ML-based diagnosis"[tiab] OR "ML recommendation"[tiab] OR "ML recommendations"[tiab] OR "ML assistance"[tiab] OR "ML support"[tiab] OR "ML advice"[tiab] OR "ML decision support"[tiab] OR "ML diagnostic systems"[tiab] OR "ML diagnosis"[tiab] OR "ML-recommendation"[tiab] OR "ML-recommendations"[tiab] OR "ML-assistance"[tiab] OR "ML-support"[tiab] OR "ML-advice"[tiab] OR "ML-decision support"[tiab] OR "ML-diagnostic systems"[tiab] OR "ML-diagnosis"[tiab] OR "ML-generated recommendation"[tiab] OR "ML-generated recommendations"[tiab] OR "ML-generated assistance"[tiab] OR "ML-generated support"[tiab] OR "ML-generated advice"[tiab] OR "ML-generated decision support"[tiab] OR "ML-generated diagnostic systems"[tiab] OR "ML-generated diagnosis"[tiab] OR "ML generated recommendation"[tiab] OR "ML generated recommendations"[tiab] OR "ML generated assistance"[tiab] OR "ML generated support"[tiab] OR "ML generated advice"[tiab] OR "ML generated decision support"[tiab] OR "ML generated diagnostic systems"[tiab] OR "ML generated diagnosis"[tiab] OR "ML-aided recommendation"[tiab] OR "ML-aided recommendations"[tiab] OR "ML-aided assistance"[tiab] OR "ML-aided support"[tiab] OR "ML-aided advice"[tiab] OR "ML-aided decision support"[tiab] OR "ML-aided diagnostic systems"[tiab] OR "ML-aided diagnosis"[tiab] OR "ML aided recommendation"[tiab] OR "ML aided recommendations"[tiab] OR "ML aided assistance"[tiab] OR "ML aided support"[tiab] OR "ML aided advice"[tiab] OR "ML aided decision support"[tiab] OR "ML aided diagnostic systems"[tiab] OR "ML aided diagnosis"[tiab] OR "DL based recommendation"[tiab] OR "DL based recommendations"[tiab] OR "DL based assistance"[tiab] OR "DL based support"[tiab] OR "DL based advice"[tiab] OR "DL based decision support"[tiab] OR "DL based diagnostic systems"[tiab] OR "DL based diagnosis"[tiab] OR "DL-based recommendation"[tiab] OR "DL-based recommendations"[tiab] OR "DL-based assistance"[tiab] OR "DL-based support"[tiab] OR "DL-based advice"[tiab] OR "DL-based decision support"[tiab] OR "DL-based diagnostic systems"[tiab] OR "DL-based diagnosis"[tiab] OR "DL recommendation"[tiab] OR "DL recommendations"[tiab] OR "DL assistance"[tiab] OR "DL support"[tiab] OR "DL advice"[tiab] OR "DL decision support"[tiab] OR "DL diagnostic systems"[tiab] OR "DL diagnosis"[tiab] OR "DL-recommendation"[tiab] OR "DL-recommendations"[tiab] OR "DL-assistance"[tiab] OR "DL-support"[tiab] OR "DL-advice"[tiab] OR "DL-decision support"[tiab] OR "DL-diagnostic systems"[tiab] OR "DL-diagnosis"[tiab] OR "DL-generated recommendation"[tiab] OR "DL-generated recommendations"[tiab] OR "DL-generated assistance"[tiab] OR "DL-generated support"[tiab] OR "DL-generated advice"[tiab] OR "DL-generated decision support"[tiab] OR "DL-generated diagnostic systems"[tiab] OR "DL-generated diagnosis"[tiab] OR "DL generated recommendation"[tiab] OR "DL generated recommendations"[tiab] OR "DL generated assistance"[tiab] OR "DL generated support"[tiab] OR "DL generated advice"[tiab] OR "DL generated decision support"[tiab] OR "DL generated diagnostic systems"[tiab] OR "DL generated diagnosis"[tiab] OR "DL-aided recommendation"[tiab] OR "DL-aided recommendations"[tiab] OR "DL-aided assistance"[tiab] OR "DL-aided support"[tiab] OR "DL-aided advice"[tiab] OR "DL-aided decision support"[tiab] OR "DL-aided diagnostic systems"[tiab] OR "DL-aided diagnosis"[tiab] OR "DL aided recommendation"[tiab] OR "DL aided recommendations"[tiab] OR "DL aided assistance"[tiab] OR "DL aided support"[tiab] OR "DL aided advice"[tiab] OR "DL aided decision support"[tiab] OR "DL aided diagnostic systems"[tiab] OR "DL aided diagnosis"[tiab] OR "model based recommendation"[tiab] OR "model based recommendations"[tiab] OR "model based assistance"[tiab] OR "model based support"[tiab] OR "model based advice"[tiab] OR "model based decision support"[tiab] OR "model based diagnostic systems"[tiab] OR "model based diagnosis"[tiab] OR "model-based recommendation"[tiab] OR "model-based recommendations"[tiab] OR "model-based assistance"[tiab] OR "model-based support"[tiab] OR "model-based advice"[tiab] OR "model-based decision support"[tiab] OR "model-based diagnostic systems"[tiab] OR "model-based diagnosis"[tiab] OR "model recommendation"[tiab] OR "model recommendations"[tiab] OR "model assistance"[tiab] OR "model support"[tiab] OR "model advice"[tiab] OR "model decision support"[tiab] OR "model diagnostic systems"[tiab] OR "model diagnosis"[tiab] OR "model-recommendation"[tiab] OR "model-recommendations"[tiab] OR "model-assistance"[tiab] OR "model-support"[tiab] OR "model-advice"[tiab] OR "model-decision support"[tiab] OR "model-diagnostic systems"[tiab] OR "model-diagnosis"[tiab] OR "model-generated recommendation"[tiab] OR "model-generated recommendations"[tiab] OR "model-generated assistance"[tiab] OR "model-generated support"[tiab] OR "model-generated advice"[tiab] OR "model-generated decision support"[tiab] OR "model-generated diagnostic systems"[tiab] OR "model-generated diagnosis"[tiab] OR "model generated recommendation"[tiab] OR "model generated recommendations"[tiab] OR "model generated assistance"[tiab] OR "model generated support"[tiab] OR "model generated advice"[tiab] OR "model generated decision support"[tiab] OR "model generated diagnostic systems"[tiab] OR "model generated diagnosis"[tiab] OR "model-aided recommendation"[tiab] OR "model-aided recommendations"[tiab] OR "model-aided assistance"[tiab] OR "model-aided support"[tiab] OR "model-aided advice"[tiab] OR "model-aided decision support"[tiab] OR "model-aided diagnostic systems"[tiab] OR "model-aided diagnosis"[tiab] OR "model aided recommendation"[tiab] OR "model aided recommendations"[tiab] OR "model aided assistance"[tiab] OR "model aided support"[tiab] OR "model aided advice"[tiab] OR "model aided decision support"[tiab] OR "model aided diagnostic systems"[tiab] OR "model aided diagnosis"[tiab] OR "computer based recommendation"[tiab] OR "computer based recommendations"[tiab] OR "computer based assistance"[tiab] OR "computer based support"[tiab] OR "computer based advice"[tiab] OR "computer based decision support"[tiab] OR "computer based diagnostic systems"[tiab] OR "computer based diagnosis"[tiab] OR "computer-based recommendation"[tiab] OR "computer-based recommendations"[tiab] OR "computer-based assistance"[tiab] OR "computer-based support"[tiab] OR "computer-based advice"[tiab] OR "computer-based decision support"[tiab] OR "computer-based diagnostic systems"[tiab] OR "computer-based diagnosis"[tiab] OR "computer recommendation"[tiab] OR "computer recommendations"[tiab] OR "computer assistance"[tiab] OR "computer support"[tiab] OR "computer advice"[tiab] OR "computer decision support"[tiab] OR "computer diagnostic systems"[tiab] OR "computer diagnosis"[tiab] OR "computer-recommendation"[tiab] OR "computer-recommendations"[tiab] OR "computer-assistance"[tiab] OR "computer-support"[tiab] OR "computer-advice"[tiab] OR "computer-decision support"[tiab] OR "computer-diagnostic systems"[tiab] OR "computer-diagnosis"[tiab] OR "computer-generated recommendation"[tiab] OR "computer-generated recommendations"[tiab] OR "computer-generated assistance"[tiab] OR "computer-generated support"[tiab] OR "computer-generated advice"[tiab] OR "computer-generated decision support"[tiab] OR "computer-generated diagnostic systems"[tiab] OR "computer-generated diagnosis"[tiab] OR "computer generated recommendation"[tiab] OR "computer generated recommendations"[tiab] OR "computer generated assistance"[tiab] OR "computer generated support"[tiab] OR "computer generated advice"[tiab] OR "computer generated decision support"[tiab] OR "computer generated diagnostic systems"[tiab] OR "computer generated diagnosis"[tiab] OR "computer-aided recommendation"[tiab] OR "computer-aided recommendations"[tiab] OR "computer-aided assistance"[tiab] OR "computer-aided support"[tiab] OR "computer-aided advice"[tiab] OR "computer-aided decision support"[tiab] OR "computer-aided diagnostic systems"[tiab] OR "computer aided recommendation"[tiab] OR "computer aided recommendations"[tiab] OR "computer aided assistance"[tiab] OR "computer aided support"[tiab] OR "computer aided advice"[tiab] OR "computer aided decision support"[tiab] OR "computer aided diagnostic systems"[tiab] OR "diagnostic assistance"[tiab] OR "diagnostic advice"[tiab] OR "decision-aid"[tiab] OR "decision-aids"[tiab] OR "diagnostic aid"[tiab] OR "diagnostic aids"[tiab] OR "automated diagnosis"[tiab] OR "automated diagnoses"[tiab] OR "decision support system"[tiab])) AND ("medicine"[tiab] OR "doctor"[tiab] OR "physician"[tiab] OR "clinician"[tiab] OR "clinic"[tiab] OR "clinics"[tiab] OR "hospital"[tiab] OR "hospitals"[tiab] OR "diagnose"[tiab] OR "diagnosis"[tiab] OR "diagnoses"[tiab] OR "health"[tiab] OR "healthcare"[tiab] OR "patient"[tiab] OR "patients"[tiab])) NOT "review"[title]

# **Extracted Data Fields**

**NOTE: The extracted values for each of these fields are available at:** [**https://osf.io/un32b/?view_only=efbeaf42a9ef4aaab45a2cde69450265**](https://osf.io/un32b/?view_only=efbeaf42a9ef4aaab45a2cde69450265)

Title

Notes (Optional)

Modality Used

Clinical Domain, Study Setting

Type of Decision-Making Task

Type of AI

What was the order of information presented

Total number of participants

Who was eligible to participate

What were the independent variables used?

How were these variables operationalized (if not obvious)?

Was Performance Measured

If performance was measured, how was it measured

If a perception was measured (trust or a proxy of trust), what was the measured perception?

What was the impact of the independent variable on this perception

If an action was measured (reliance or a proxy of reliance), what was the measured action?

What was the impact of the independent variable on this action

What was the impact of the independent variable on performance
